# Supplementary material for: Sensory cortex plasticity supports auditory social learning
Source: Nat Commun. 2023 Sep 20;14:5828. doi: 10.1038/s41467-023-41641-8 (PMC10511464; doi:10.1038/s41467-023-41641-8)
Supplement: Supplementary file 1 — Supplementary Information [file 41467_2023_41641_MOESM1_ESM.pdf]

Title **Sensory cortex plasticity supports auditory social learning**

Author List Nihaad Paraouty*[1], Justin D. Yao[2], Léo Varnet[3], Chi-Ning Chou[4,5], SueYeon Chung[1,4], Dan H. Sanes[1,6,7,8]

Affiliations
[1]Center for Neural Science
New York University, New York, NY 10003
[2]Department of Otolaryngology
Rutgers University, New Brunswick, NJ, 08901
[3]Laboratoire des Systèmes Perceptifs, UMR 8248
École Normale Supérieure, Paris 75005
[4]Center for Computational Neuroscience
Flatiron Institute, Simons Foundation
[5]School of Engineering & Applied Sciences
Harvard University, Cambridge, MA 02138
[6]Department of Psychology, New York University, New York, NY 10003
[7]Department of Biology, New York University, New York, NY 10003
[8]Neuroscience Institute, NYU Langone Medical Center, New York, NY 10003

Supplementary Figures 1-7

Supplementary Tables 1-2

Supplementary Note 1

Supplementary Figures

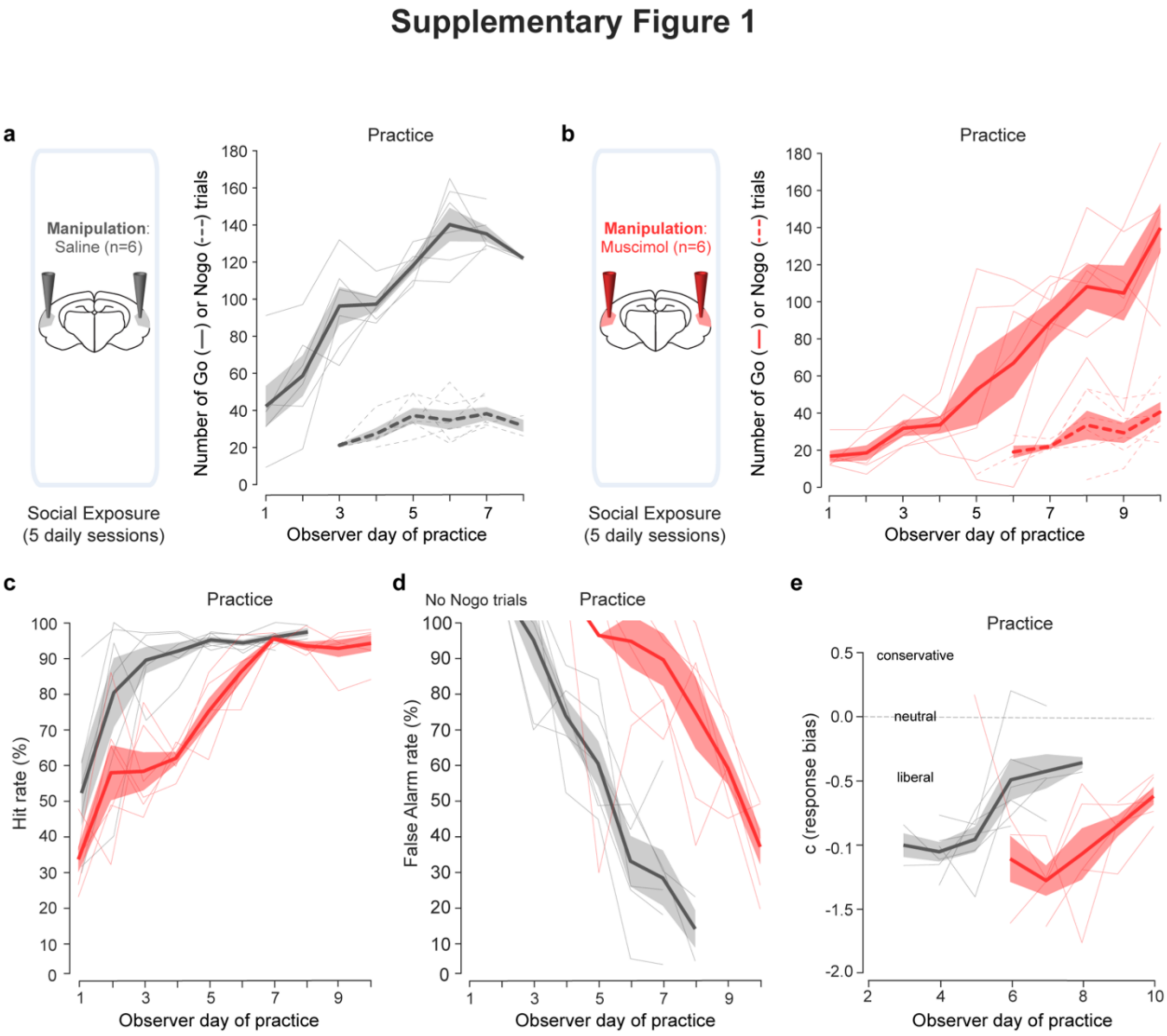

Supplementary Figure 1: Behavioral performance of saline- and muscimol-infused social observers.

a. Number of Go (gray full lines) and Nogo trials (gray dashed lines) initiated by saline-infused *social observers* during practice. Thin lines denote individual animals; thick lines and transparent area denote mean ± SE. **b**. Number of Go (red full lines) and Nogo trials (red dashed lines) initiated by muscimol-infused *social observers* during practice. No significant difference between the performance of the demonstrators in the 2 groups (Wilcoxon rank sum test, two-sided, $X^2(1)=2.04$, $p=0.15$). **c**. Hit rate of saline- (gray) and muscimol-infused (red) *social observers*. **d**. False Alarm rate in practice sessions with > 15 Nogo trials. **e**. Response bias in practice sessions with > 15 Nogo trials.

Supplementary Figure 2

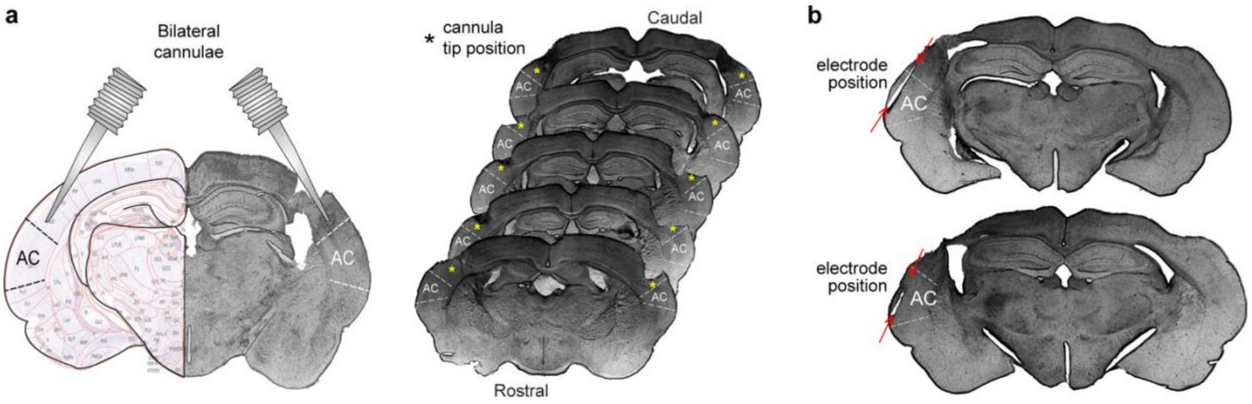

Supplementary Figure 2: Anatomically confirmed cannulae and electrode tracks in auditory cortex

a. Schematic of cannula position in auditory cortex (AC) using the gerbil brain atlas (left; Radtke-Schuller et al., 2016) and a representative coronal section from one implanted animal (right). Yellow asterisks indicate cannula tip positions. **b**. Representative coronal sections from two electrode implanted animals. Red arrow indicates electrode tracks through AC.

Supplementary Figure 3

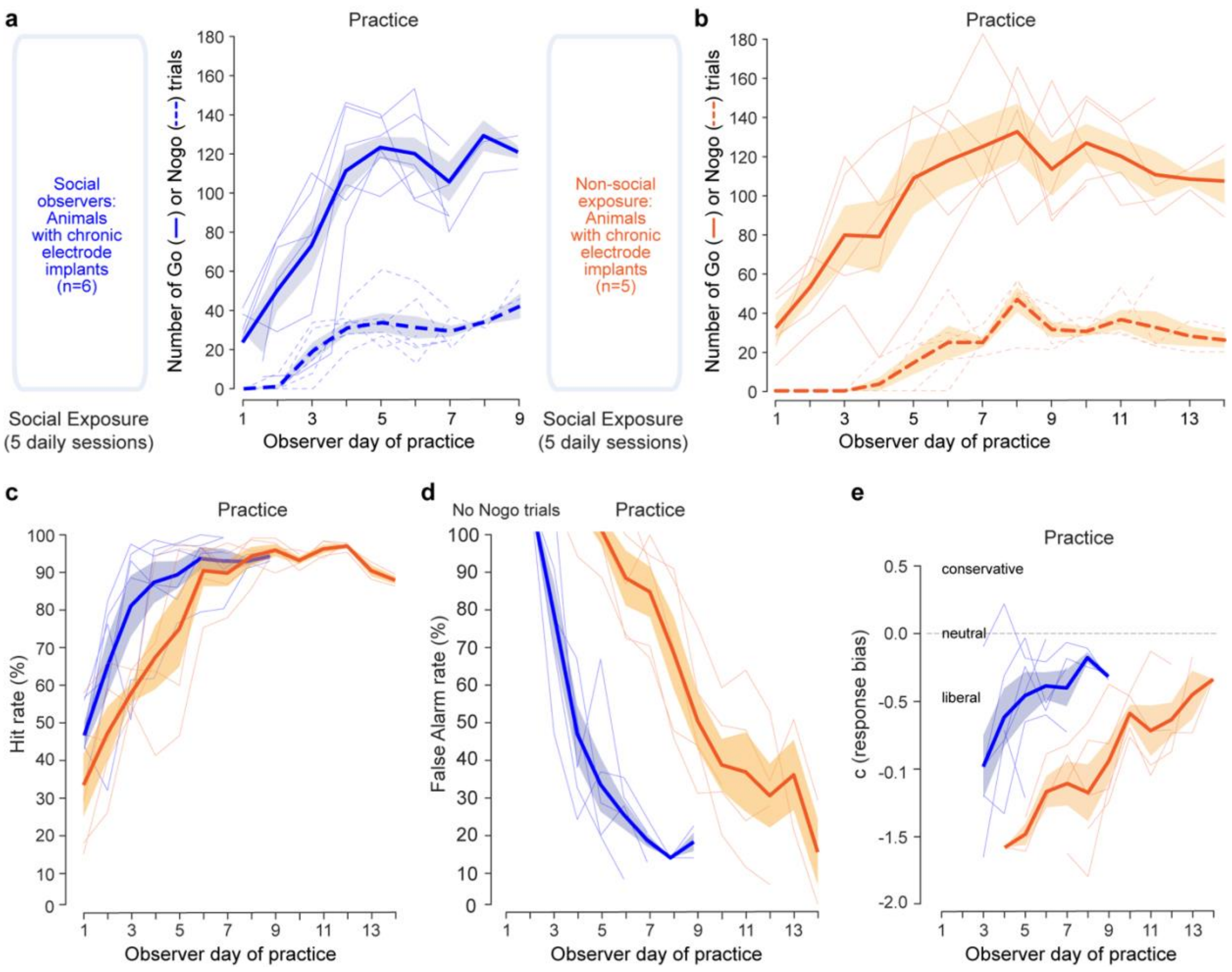

Supplementary Figure 3: Behavioral performance of animals with social exposure as compared to those with non-social exposure

a. Number of Go (blue full lines) and Nogo trials (blue dashed lines) performed by *social observers* during practice sessions. Thin lines denote individual animals; thick lines and transparent area denote mean ± SE. **b**. Number of Go (orange full lines) and Nogo trials (orange dashed lines) performed by *non-social exposure* animals during practice sessions. The experimenter-triggered exposure sessions (in panel b) matched those of the demonstrator animals (in panel a). **c**. Hit rate of *social observers* (blue lines) and *non-social exposure* animals (orange lines). **d**. False Alarm rate in practice sessions with > 15 Nogo trials. **e**. Response bias in practice sessions with > 15 Nogo trials.

Supplementary Figure 4

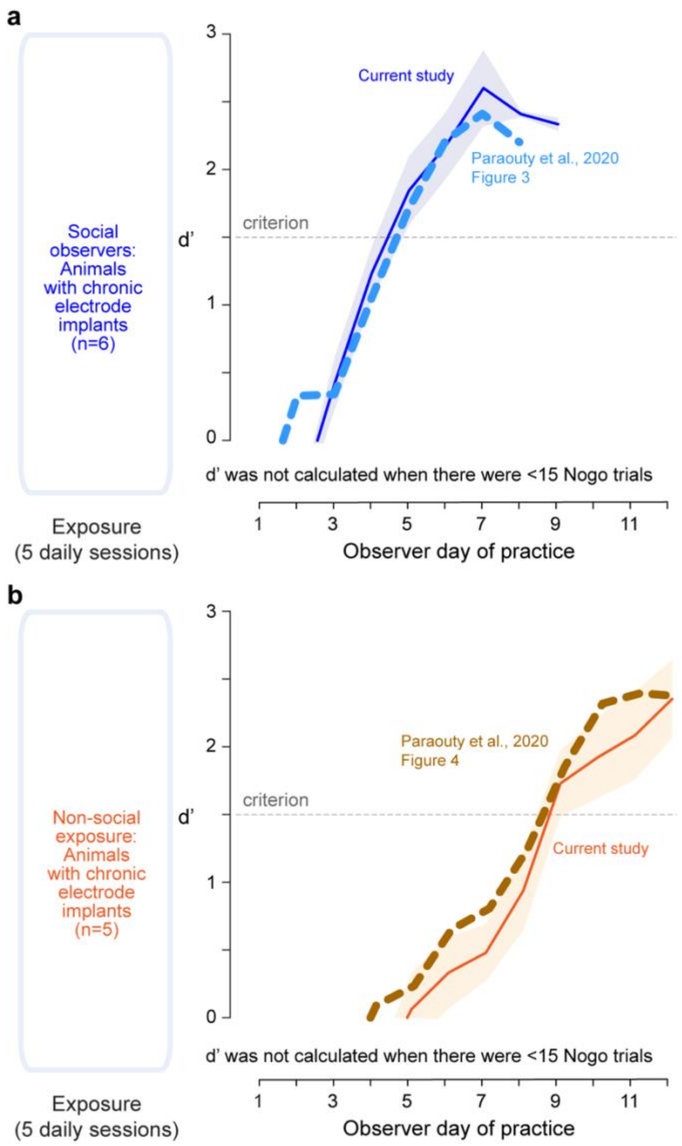

Supplementary Figure 4: No behavioral performance difference between implanted and non-implanted animals

a. Behavioral d' performance of implanted *social observers* in practice sessions with >15 Nogo trials (blue solid line; mean ± standard error). The mean behavioral d' performance of non-implanted *social observers* tested in a previous study (Paraouty et al., 2020) is shown for comparison (cyan dashed line; mean). No significant difference was observed (Steel-Dwass comparison, two-sided, p=0.999). **b**. Behavioral d' performance of implanted *non-social exposure* animals in practice sessions with >15 Nogo trials (orange solid line; mean ± standard error). The mean behavioral d' performance of non-implanted *non-social exposure* animals tested previously (Paraouty et al., 2020) is shown for comparison (brown dashed line; mean). No significant difference was observed (p=0.934). No d' was computed when the observers initiated <15 Nogo trials in the practice sessions.

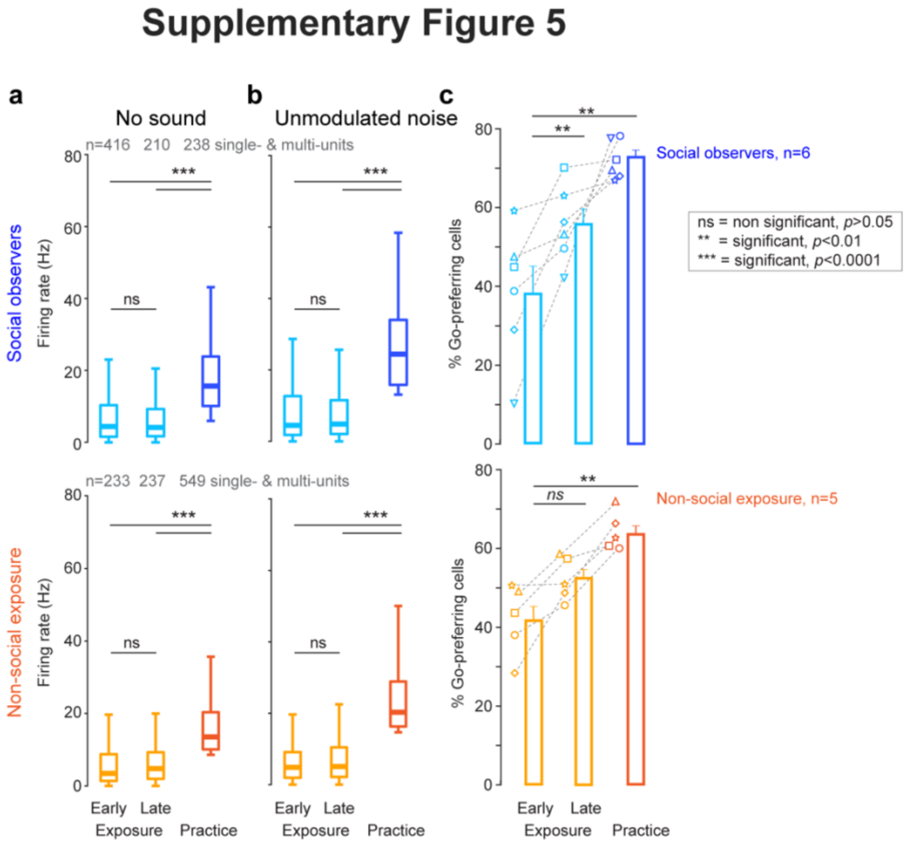

Supplementary Figure 5: Social exposure selectively increases the percentage of Go-preferring cells

a. Spontaneous firing rate (calculated 200 ms before sound onset) for all single- and multi-units during early and late exposure and practice sessions for *social observers* (blue shades) and *non-social exposure* animals (orange shades). The central mark indicates median, the bottom and top edges of the box indicate the 25th and 75th percentiles, the whiskers extend to the most extreme minimum and maximum points not considered outliers. No significant change in spontaneous firing rate was found between early and late exposure sessions in both groups (Tukey-Kramer HSD comparisons; corrected for multiple comparisons; p=0.322; p=0.175). However, from exposure to practice, a significant increase in firing rate was observed in both groups (both p<0.0001). **b**. Firing rate to unmodulated AM portions of stimuli (calculated from 200 to 400 ms of sound onset). No significant change in spontaneous firing rate was found between early and late exposure sessions in both groups (Tukey-Kramer HSD comparisons; corrected for multiple comparisons; p=0.245; p=0.411). However, from exposure to practice, a significant increase in firing rate was observed in both groups (both p<0.0001). **c**. The firing rate response to the AM portion of the signal was further examined and cells that responded with a higher increase in firing rate to the Go AM (response to Go - spontaneous) as compared to the Nogo AM (response to Nogo - spontaneous) were classified as Go-preferring cells. In contrast, cells which showed a higher increase in firing rate to the Nogo AM as compared to the Go AM were classified as Nogo-preferring cells. For all animals, the percentage of Go-preferring cells increased between early exposure and practice (each symbol represents data from a single animal; Steel-Dwass comparisons, two-sided; *social observers*, p=0.002; *non-social exposure* animals, p=0.0079). For *social observers*, a significant increase in the percentage of Go cells was also observed between early and late exposure (p=0.002). However, the *non-social exposure* animals did not display a significant difference between early and late exposure (p=0.841).

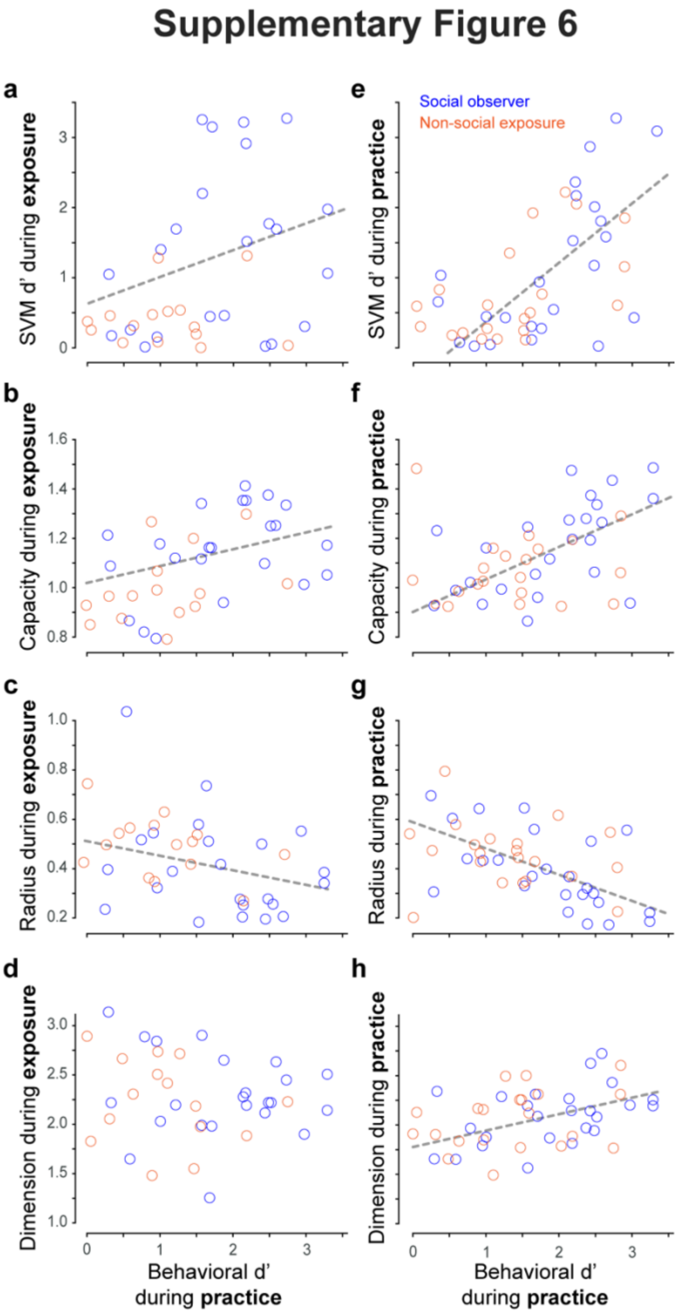

Supplementary Figure 6: Correlation between neural population measures during exposure or practice and behavioral performance during practice

a. Significant correlation between population neural d' during exposure and behavioral d' during practice (Pearson's; r=0.37, *p*=0.002). **b**. Significant correlation between manifold capacity during exposure and behavioral d' during practice (r=0.49, *p*=0.002). **c**. Significant correlation between manifold radius during exposure and behavioral d' during practice (r=-0.40, *p*=0.012). **d**. No significant correlation between manifold dimension during exposure and behavioral d' during practice r=-0.13, *p*=0.43). **e**. Significant correlation between population neural d' during practice and behavioral d' during practice (r=0.63, *p*<0.0001).

f. Significant correlation between manifold capacity during exposure and behavioral d' during practice (r=0.39, p=0.007). **g**. Significant correlation between manifold radius during exposure and behavioral d' during practice (r=-0.48, p=0.0007). **h**. Significant correlation between manifold dimension during exposure and behavioral d' during practice r=0.39, p=0.007).

Supplementary Figure 7

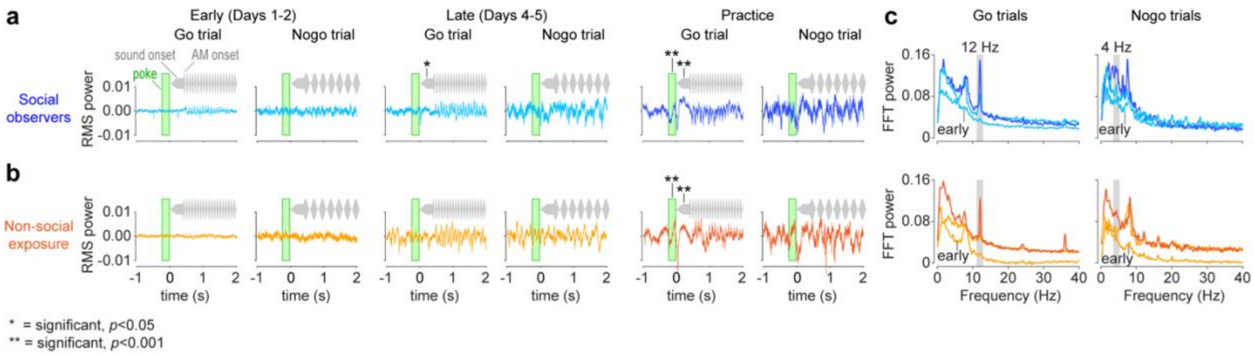

Supplementary Figure 7: Social exposure leads to a significant increase in stimulus-evoked power

a. Left panel: mean stimulus-evoked RMS power fluctuations in response to Go and Nogo trials during early exposure for a *social observer* (blue) aligned with demonstrator poke (green panel). Induced power is shown for 2 frequency bands: a low frequency (6-20 Hz; thick lines) and a high frequency (20-80 Hz; thin lines). Middle panel: late exposure sessions. Right panel: practice sessions with behavioral d' > 1.5. **b**. Mean stimulus-evoked RMS power fluctuations in response to Go and Nogo trials for a *non-social exposure* animal (orange) aligned with the experimenter-triggered poke. During early exposure, no significant change from baseline (zero) was observed for all *social observers* and all *non-social exposure* animals at sound onset (from 0 to 0.4 sec; two-sample t-tests on individual animals for the Go stimuli, all $p>0.05$; see Supplementary Table 2). During late exposure, a significant change from baseline was present at sound onset for *social observers* only (all $p<0.05$ for *social observers* and all $p>0.05$ for *non-social exposure* animals). During practice, significant changes from baseline were found for both *social observers* and *non-social exposure* animals at sound onset, as well as prior to sound onset (i.e., during the poke = -0.3 to -0.1 sec; all $p<0.05$). **c**. Fast-Fourier transform (FFT) computed for the AM portion of the stimuli (pale lines: exposure; darker line: practice). The mean peak amplitude at 12 and 4 Hz of the FFT increased significantly with exposure for both *social observers* and *non-social exposure* animals (mixed-model ANOVA, early vs. late exposure; $F(1,36)=39.9$, $p<0.0001$). No significant group difference was observed ($F(1,36)=3.3$, $p=0.078$). A significant interaction between group and exposure period was present ($F(1,36)=4.5$, $p=0.039$). During practice, the mean peak FFT amplitude increased significantly as compared to both exposure periods for both groups ($F(1,54)=54.2$, $p<0.0001$).

Supplementary Tables

Supplementary Table 1: Two-tailed t-test for individual animals on induced power fluctuations

Induced power assesses the oscillatory activity not necessarily time-locked to the AM stimuli.

p values corrected for multiple comparisons	Induced power at **poke**<br><br>(*p* value for Low; High frequency band)			Induced power at **reward**<br><br>(*p* value for Low frequency band)		
	Early Exposure	Late Exposure	Practice	Early Exposure	Late Exposure	Practice
Social observer 1	p=0.76; p=0.84	p<0.0001; p<0.0001	p<0.0001; p<0.0001	p=0.001	p<0.0001	p<0.0001
Social observer 2	p=0.52; p=0.68	p<0.0001; p<0.0001	p<0.0001; p<0.0001	p=0.021	p<0.0001	p<0.0001
Social observer 3	p=0.29; p=0.67	p<0.0001; p<0.0001	p<0.0001; p<0.0001	p=0.037	p<0.0001	p<0.0001
Social observer 4	p=0.37; p=0.29	p<0.0001; p<0.0001	p<0.0001; p<0.0001	p=0.004	p<0.0001	p<0.0001
Social observer 5	p=0.63; p=0.22	p<0.0001; p<0.0001	p<0.0001; p<0.0001	p=0.011	p=0.003	p<0.0001
Social observer 6	p=0.10; p=0.22	p=0.001; p=0.003	p<0.0001; p<0.0001	p=0.008	p=0.001	p<0.0001
Non-social animal 1	p=0.87; p=0.98	p=0.06; p=0.49	p<0.0001; p<0.0001	p=0.18	p=0.43	p<0.0001
Non-social animal 2	p=0.67; p=0.86	p=0.08; p=0.67	p=0.009; p=0.042	p=0.88	p=0.51	p<0.0001
Non-social animal 3	p=0.42; p=0.77	p=0.05; p=0.42	p=0.003; p=0.008	p=0.47	p=0.39	p<0.0001
Non-social animal 4	p=0.52; p=0.88	p=0.33; p=0.75	p=0.020; p<0.0001	p=0.76	p=0.44	p<0.0001
Non-social animal 5	p=0.62; p=0.88	NaN	NaN	p=0.51	NaN	NaN

Supplementary Table 2: Two-tailed t-test for individual animals on stimulus-evoked power fluctuations

Evoked power assesses the precise phase-locking of AC activity to the auditory AM stimuli across trials.

p values corrected for multiple comparisons	Stimulus-evoked power at sound onset		
	Early Exposure	Late Exposure	Practice
Social observer 1	p=0.10	p=0.02	p<0.0001
Social observer 2	p=0.08	p=0.018	p<0.0001
Social observer 3	p=0.19	p=0.025	p<0.0001
Social observer 4	p=0.77	p=0.007	p<0.0001
Social observer 5	p=0.37	p=0.01	p<0.0001
Social observer 6	p=0.27	p=0.04	p<0.0001
Non-social animal 1	p=0.52	p=0.51	p<0.0001
Non-social animal 2	p=0.29	p=0.55	p<0.0001
Non-social animal 3	p=0.10	p=0.08	p<0.0001
Non-social animal 4	p=0.22	p=0.03	p<0.0001
Non-social animal 5	p=0.49	NaN	NaN

Supplementary Note 1

Statistical comparisons were performed as followed:

For Figure 1:
To assess the impact of muscimol infusions, we first used Kruskal-Wallis rank sum test to compare the number of days to criterion d' for the following groups, followed by post hoc nonparametric comparisons for all pairs with the Steel-Dwass method:

- Saline-infused *social observers,*
- Muscimol-infused *social observers,*
- Non-visual *social observers* (from Figure 3 in Paraouty et al., 2020),
- Muscimol-infused *social observers* - 1 day,
- Muscimol-infused *social observers* - 2 days.

Next, to compare the performance of muscimol-infused *social observers* with all our previous control paradigms (from Paraouty et al., 2020), we used the same approach described above, and compared the following groups:

- Muscimol-infused *social observers,*
- Controls for all exposure (from Figure 2c in Paraouty et al., 2020),
- Controls for social exposure (from Figure 2a in Paraouty et al., 2020),
- Controls for cage exposure (from Figure 2b in Paraouty et al., 2020).

For Figure 2:
To assess the impact of social vs. non-social exposure, as well as electrode implantation, we first used Kruskal-Wallis rank sum test to compare the number of days to criterion d' for the following groups, followed by post hoc nonparametric comparisons for all pairs with the Steel-Dwass method:

- Electrode-implanted *social observers,*
- Electrode-implanted *non-social exposure* animals,
- Non-implanted *social observers* (from Figure 3 in Paraouty et al., 2020),
- Non-implanted *non-social exposure* animals (from Figure 4b in Paraouty et al., 2020).

For Figure 3:
To compare Firing rate, we used mixed-model ANOVA's followed by post hoc Tukey-Kramer HSD comparisons with corrected alpha values.
Groups: social observers vs. non-social exposure animals.
Epochs: Early exposure, Late exposure and Practice.
Firing rate variables: Spontaneous, Unmodulated noise, and Modulated noise.

To compare the coefficient of variation, we first used Kruskal-Wallis test, followed by post hoc nonparametric comparisons for all pairs with the Steel-Dwass method. The same method was used for vector strength comparisons.

To compare the neural d' for all single-units, we first used Kruskal-Wallis test, followed by post hoc nonparametric comparisons for all pairs with the Steel-Dwass method.

To compare the neural d' for single-units from the same recording sites, we used two-sample t-tests to test for significant differences, and computed a Likelihood ratio Chi square to assess the changes in the 2 groups.

We calculated Pearson's *r* and its associated *p* value to analyze the relationship between median change in neural d' for all single-units and days to reach behavioral criterion d'.

For Figure 4:
To look at population neurometrics, we computed mixed-model ANOVA's for each of the following variables:

- SVM neural d',
- Manifold capacity,
- Manifold radius,
- Manifold dimension.

For Figure 5:
For each individual animal, we used two-samples t-tests to analyze the LFP average differences as compared to baseline in the 2 regions of interest:

- Poke, and
- Reward.

Supplementary References

Radtke-Schuller S, Schuller G, Angenstein F, Grosser OS, Goldschmidt J, Budinger E (2016) Brain atlas of the Mongolian gerbil (Meriones unguiculatus) in CT/MRI-aided stereotaxic coordinates. Brain Struct Funct 221 Suppl 1:1-272.

Paraouty N, Charbonneau JA, Sanes DH (2020) Social learning exploits the available auditory or visual cues. Sci Rep, 10:14117.
