# Supplementary material for: Sensory cortex plasticity supports auditory social learning
Source: Nat Commun. 2023 Sep 20;14:5828. doi: 10.1038/s41467-023-41641-8 (PMC10511464; doi:10.1038/s41467-023-41641-8)
Supplement: Supplementary file 3 — Reporting Summary [file 41467_2023_41641_MOESM3_ESM.pdf]

# nature portfolio

Corresponding author(s): Nihaad PARAOUTY

Last updated by author(s): 02 August 2023

# Reporting Summary

Nature Portfolio wishes to improve the reproducibility of the work that we publish. This form provides structure for consistency and transparency in reporting. For further information on Nature Portfolio policies, see our Editorial Policies and the Editorial Policy Checklist.

## Statistics

For all statistical analyses, confirm that the following items are present in the figure legend, table legend, main text, or Methods section.

| n/a | Confirmed | |
|---|---|---|
| ☐ | ☒ | The exact sample size (*n*) for each experimental group/condition, given as a discrete number and unit of measurement |
| ☐ | ☒ | A statement on whether measurements were taken from distinct samples or whether the same sample was measured repeatedly |
| ☐ | ☒ | The statistical test(s) used AND whether they are one- or two-sided<br>*Only common tests should be described solely by name; describe more complex techniques in the Methods section.* |
| ☐ | ☒ | A description of all covariates tested |
| ☐ | ☒ | A description of any assumptions or corrections, such as tests of normality and adjustment for multiple comparisons |
| ☐ | ☒ | A full description of the statistical parameters including central tendency (e.g. means) or other basic estimates (e.g. regression coefficient) AND variation (e.g. standard deviation) or associated estimates of uncertainty (e.g. confidence intervals) |
| ☐ | ☒ | For null hypothesis testing, the test statistic (e.g. *F*, *t*, *r*) with confidence intervals, effect sizes, degrees of freedom and *P* value noted<br>*Give P values as exact values whenever suitable.* |
| ☒ | ☐ | For Bayesian analysis, information on the choice of priors and Markov chain Monte Carlo settings |
| ☒ | ☐ | For hierarchical and complex designs, identification of the appropriate level for tests and full reporting of outcomes |
| ☐ | ☒ | Estimates of effect sizes (e.g. Cohen's *d*, Pearson's *r*), indicating how they were calculated |

*Our web collection on statistics for biologists contains articles on many of the points above.*

## Software and code

Policy information about availability of computer code

| | |
|---|---|
| Data collection | All behavioral data were collected using custom MATLAB (MathWorks) scripts developed by Daniel Stolzberg (https://github.com/dstolz/epsych) and Tucker-Davis Technologies hardware (RZ6 multifunction processor). In-vivo electrophysiological recordings were collected using a wireless recording system (headstage and receiver, W64, Triangle Biosystems). Analog signals were amplified and digitized at a sampling frequency of 24414 Hz and transmitted to a digital signal processor (Tucker-Davis Technologies; 16-channel recordings: TB32 to RZ5; 64-channel recordings: PZ5 to RZ2), then sent to a PC for storage and post-processing using Tucker-Davis Technologies Synapse Suite (https://www.tdt.com/component/synapse-software/). |
| Data analysis | All behavioral and physiological data were analyzed using custom MATLAB (MathWorks) or Python scripts. Statistical analyses were performed using JMP Pro 14.0 and 16.0 (SAS) on a Mac platform, and custom-written MATLAB scripts (R2021a). Open-source spike sorting packages were used to extract and cluster spike waveforms (16-channel recordings: UltraMegaSort 2000; 64-channel recordings: KiloSort, Pachitariu et al., 2016; https://github.com/cortex-lab/KiloSort). Manual inspection of spike waveforms was conducted in Phy 2 (Rossant et al., 2016; https://github.com/cortex-lab/phy). Custom scripts for data analysis have been deposited on an NYU repository (NYU app box) and can be accessed here: https://nyu.box.com/s/rv9m4ionulfk42nkbamff1h706xaeko7 in the folder: Matlab code. |

For manuscripts utilizing custom algorithms or software that are central to the research but not yet described in published literature, software must be made available to editors and reviewers. We strongly encourage code deposition in a community repository (e.g. GitHub). See the Nature Portfolio guidelines for submitting code & software for further information.

## Data

Policy information about availability of data

All manuscripts must include a data availability statement. This statement should provide the following information, where applicable:

- Accession codes, unique identifiers, or web links for publicly available datasets
- A description of any restrictions on data availability
- For clinical datasets or third party data, please ensure that the statement adheres to our policy

The data generated in this study have been deposited on an NYU repository (NYU app box) and can be accessed here: https://nyu.box.com/s/rv9m4ionulfk42nkbamff1h706xaeko7. Source data files for Figures 1-5 are provided separately. Raw electrophysiological data can be shared upon request to the first author (np64@nyu.edu; ongoing further computational analysis by co-authors).

## Human research participants

Policy information about studies involving human research participants and Sex and Gender in Research.

| | |
|---|---|
| Reporting on sex and gender | Not Applicable |
| Population characteristics | Not Applicable |
| Recruitment | Not Applicable |
| Ethics oversight | Not Applicable |

Note that full information on the approval of the study protocol must also be provided in the manuscript.

# Field-specific reporting

Please select the one below that is the best fit for your research. If you are not sure, read the appropriate sections before making your selection.

☒ Life sciences ☐ Behavioural & social sciences ☐ Ecological, evolutionary & environmental sciences

For a reference copy of the document with all sections, see nature.com/documents/nr-reporting-summary-flat.pdf

# Life sciences study design

All studies must disclose on these points even when the disclosure is negative.

| | |
|---|---|
| Sample size | Sample size was determined based on previous behavioral studies from our laboratory and associated power analyses that provided a minimum number of subjects required per group to establish significance (see Paraouty et al., 2020; 2021). |
| Data exclusions | No animals were removed from any of the behavioral or in-vivo electrophysiology experiments. In-vivo electrophysiology data was not included if the recording probes were not targeting core auditory cortex, or if neural responses did not meet specific auditory response criteria (specified in the Methods). All exclusion criteria were established prior to data collection. |
| Replication | All experiments were replicated in multiple animals. The numbers of animals per group are detailed in the manuscript or in the figure legends. The behavioral findings presented here represent a replication of our previous results (see Paraouty et al., 2020). All attempts at replication were successful. |
| Randomization | All animals within a litter were randomly assigned to one of the behavioral groups. For all behavioral and in-vivo electrophysiology experiments, similar number of male and female animals were used. |
| Blinding | For all behavioral and in-vivo electrophysiology experiments, blinding was not relevant as identical criteria, analysis pipeline and unbiased statistical tests were used. |

# Reporting for specific materials, systems and methods

We require information from authors about some types of materials, experimental systems and methods used in many studies. Here, indicate whether each material, system or method listed is relevant to your study. If you are not sure if a list item applies to your research, read the appropriate section before selecting a response.

## Materials & experimental systems

| n/a | Involved in the study |
|---|---|
| ☒ | ☐ Antibodies |
| ☒ | ☐ Eukaryotic cell lines |
| ☒ | ☐ Palaeontology and archaeology |
| ☐ | ☒ Animals and other organisms |
| ☒ | ☐ Clinical data |
| ☒ | ☐ Dual use research of concern |

## Methods

| n/a | Involved in the study |
|---|---|
| ☒ | ☐ ChIP-seq |
| ☒ | ☐ Flow cytometry |
| ☒ | ☐ MRI-based neuroimaging |

# Animals and other research organisms

Policy information about studies involving animals; ARRIVE guidelines recommended for reporting animal research, and Sex and Gender in Research

| | |
|---|---|
| Laboratory animals | Male and female Mongolian gerbils (Meriones unguiculatus) were used. Animals were weaned at postnatal day (P) 30 from commercial breeding pairs (Charles River), and littermates were caged together, but separated by sex, and maintained in a 12 h light/dark cycle. All animals involved in the study were older than P60. |
| Wild animals | No wild animals were used in this study. |
| Reporting on sex | Care was taken to ensure similar distribution of male and female animals in all experimental groups. No sex differences are expected (see Paraouty et al., 2020; no significant difference was found between male and female gerbils in terms of number of days to reach criterion d', when combined across groups or within each group (all p >0.05). Hence, both male and female gerbils are used in this study for the behavioral and electrophysiological analysis. |
| Field-collected samples | The study did not involve samples collected from the field. |
| Ethics oversight | All procedures related to the maintenance and use of animals were approved by the Institutional Animal Care and Use Committee at New York University, and all experiments were performed in accordance with the relevant guidelines and regulations. |

Note that full information on the approval of the study protocol must also be provided in the manuscript.
